# Novel Porous Nitrogen Doped Graphene/Carbon Black Composites as Efficient Oxygen Reduction Reaction Electrocatalyst for Power Generation in Microbial Fuel Cell

**DOI:** 10.3390/nano9060836

**Published:** 2019-06-01

**Authors:** Yuan Liu, Zhimei Liu, Hong Liu, Meiling Liao

**Affiliations:** 1 Key Laboratory of Reservoir Aquatic Environment, Chinese Academy of Sciences, Chongqing 400714, China; liaomeiling@cigit.ac.cn; 2 Chongqing Institute of Green and Intelligent Technology, Chinese Academy of Sciences, Chongqing 400714, China; liuzhimei@mails.ucas.edu.cn

**Keywords:** template-like method, nitrogen-doped graphene, carbon black, oxygen reduction reaction, microbial fuel cell

## Abstract

To improve the power generation of a microbial fuel cell (MFC), a porous nitrogen-doped graphene/carbon black (NG/CB) composite as efficient oxygen reduction reaction (ORR) electrocatalyst was successfully synthesized by pyrolyzing graphene oxide (GO) encapsulated CB with cetyltrimethyl ammonium bromide as a bridge. This concept-to-proof synthesis can be considered as a template-like method. Based on this method, one composite named as NG/CB-10 was acquired using the optimized GO-to-CB mass ratio of 10:1. Electrochemical tests demonstrate that NG/CB-10 can catalyze ORR in neutral-pH medium through a four-electron pathway with positively shifted the onset potential, the enhanced current density and reduced charge transfer resistance. CB addition also prolongs the stability of NG/CB-10. The enhancement in electrochemical performance of NG/CB-10 was attributed to the enlarged surface area, abundant mesopores and high content of pyridinic nitrogen. The maximum power density of MFC equipping NG/CB-10 as cathode electrocatalyst reached 936 mW·m^−2^, which was 26% higher than that of NG and equal to that of platinum/carbon. The cost of NG/CB-10 was reduced by 25% compared with that of NG. This work provides a novel method to synthesize promising ORR electrocatalyst for MFC in the future.

## 1. Introduction

Oxygen reduction reaction (ORR) has become a dominant cathode reaction in microbial fuel cells (MFCs) due to its easy operation and potential for large-scale application [1,2,3,4]. A four-electron (4e^−^) transfer pathway of ORR with H_2_O as final product facilitates the MFCs to achieve high energy output. Among the numerous electrocatalysts, nitrogen-doped graphene (NG) has been verified to be efficient for the 4e^−^ ORR [5,6,7,8]. NG has been intensively investigated as cathode electrocatalyst in MFCs or chemical fuel cells since its first synthesis as an electrocatalyst for ORR in alkaline solution [9,10,11,12,13]. Heteroatom doping into graphene sheets raises the Fermi energy and provides abundant highly active catalytic sites, which significantly improve the electrocatalytic properties for ORR [14,15,16,17]. NG faces two main challenges despite high ORR activity. On one hand, contrary to pristine graphene, the doping of heteroatom into graphene sheets causes structural defects. These defects provide ORR active sites but severely deteriorate the electrical properties of NG [16,18]. On the other hand, restacking of graphene sheets via van der Waals forces reduces the active surface area and accessibility of electrocatalytically active sites, which result in decreased ORR activity [15,19,20]. The restacking of graphene sheets also causes porosity reduction. The mass transport of reactants and products becomes deteriorated, which leads to severe concentration polarization and loss of electromotive force [21,22,23]. Therefore, the suppressed electrocatalytic activity of NG depresses performance of energy-conversion cells.

Assembling NG into three-dimensional (3D) porous architectures has become one of the promising approaches to avoid above-mentioned challenges. Template-assisted synthesis is a common method to obtain 3D NG, in which silica spheres and nickel foam are widely used as the hard templates [24,25,26,27]. The hard-template method is often known as the sacrificial support method. This method can efficiently synthesize 3D NG with high surface area and enhanced electrocatalytic activity, but the post-treatment removal of these hard templates complicates the fabrication procedures. Hard templates are usually not cost-effective. Moreover, acid leaching in the post-treatment can also cause the loss of active sites of NG. The soft-template method seems to be an alternative to the hard-template method. Taking advantage of cheaper surfactants as soft templates, mesoporous NG can be easily obtained by sintering the mixture of templates and carbon precursors [28,29,30,31]. These soft templates can be readily removed during the sintering procedure under high temperature. In our previous study, a porous 3D NG was synthesized by using cetyltrimethyl ammonium bromide (CTAB) micelles as a soft template and showed enhanced porosity and ORR activity in a neutral-pH medium [23]. Electrostatic force between negatively and positively charged graphene oxide (GO) and CTAB micelle, respectively, was utilized to form porous NG structure. Nevertheless, unlike the hard template, the soft template demonstrated a structure that was prone to deformation, resulting in decreased porosity of the final product. Hence, the surface area of 3D NG made from a soft template is often smaller than that from the hard template.

Apart from using templates to synthesize 3D NG, introducing “spacer” phases between graphene sheets to form sandwich-type structures is also effective [20,32,33,34]. Commonly, one-dimensional carbon nanotubes (CNTs) are introduced into two-dimensional graphene materials to construct 3D graphene-CNT composite materials [15,16,35,36,37]. Covalently bonded graphene and CNT hybrid material can adjust their electronic properties for application in energy-conversion cells [38,39]. Similarly, NG-CNT composite can be achieved by a pyrolyzing mixture of GO, CNT, and nitrogen-rich source. This composite showed enhanced ORR activity and superior durability in alkaline condition [15,16,37]. Some disadvantages of the “spacer” approach must be carefully considered. The amount of CNT is often higher than that of graphene to obtain a well-structured 3D graphene-CNT composite. Both CNT and graphene are expensive, so that the high cost of the final product may be unsuitable for MFCs. Although the restacking of graphene sheets can be alleviated by introducing spacer, some active sites are still buried in the inner phase of sandwich-type structures and cannot serve for ORR. 

**Figure 1:**
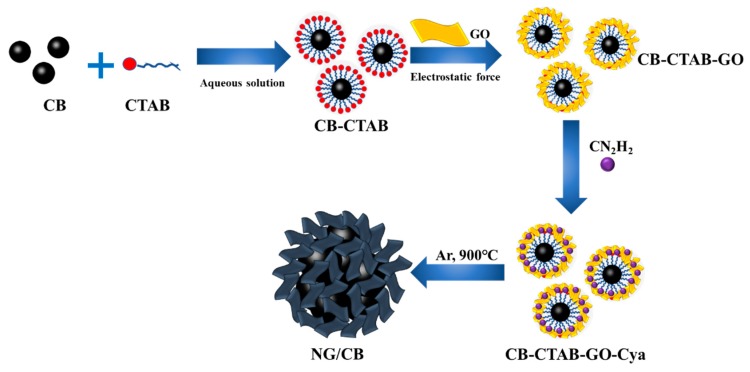
Schematic route of synthesis of nitrogen-doped graphene/carbon black -x electrocatalysts.

Consequently, these previous studies stimulated us to propose a new synthesis route to achieve NG/carbon composite with increased porosity and superior ORR activity. In the new route (Figure 1), a cheap, conductive, and hydrophobic carbon material, for example, carbon black (CB) with high surface area, was introduced as the backbone, and CTAB was used as a bridge. The hydrophobic end of CTAB was adsorbed on CB, leaving the hydrophilic end in the aqueous phase. The hydrophilic end of CTAB then reacted with GO sheet via electrostatic force, which resulted in the formation of a GO encapsulated CB composite. Followed by the addition of nitrogen-rich source and high-temperature treatment, an nitrogen-doped graphene/carbon black (NG/CB) composite with NG as an outside catalytic layer and CB as inside supporting layer can be achieved. Similar to typical core-shell structure, the outside layer of NG can serve as ORR active sites, and the inside CB can function as electricity collector. The thickness of the outside layer can be easily controlled by adjusting the amount of GO precursor, and the amount of graphene can be minimized to cut down the cost of the final product. Moreover, the high surface area of CB can improve the porosity of the NG/CB composite. The introduction of CB is able to alleviate the stacking of graphene sheets and improve the electrical performance of NG. The new synthesis can be named as the template-like method. To our knowledge, this new method for synthesis of NG/CB composite and application in MFCs has not yet been reported. 

In this work, we first synthesized the NG/CB composite materials. The ORR activity and other electrochemical properties of the NG/CB composites in neutral-pH medium were then systematically evaluated. The physicochemical properties of the composite were carefully estimated, including porosity information, elemental composition, and crystalline structure. The NG/CB composites were also applied as cathode electrocatalyst in MFCs to evaluate the electricity-generation ability.

## 2. Materials and Methods

### 2.1. Synthesis of NG/CB Composites

First, 50 mg GO (Suzhou TANFENG graphene Tech Co., Ltd., Suzhou, China) was ultrasonically dispersed in deionized water for 30 min to obtain a concentration of 2 mg·mL^−1^. CB (Vulcan XC-72, Cabot) was added into 0.1 mg·mL^−1^ of CTAB solution to reach a mass ratio of 5:1 (CB:CTAB), and the suspension was ultrasonicated for 30 min to obtain well-dispersed CB/CTAB solution. The two suspensions with certain volumes were carefully mixed to get a mass ratio of GO and CB from 1:1 to 20:1 under continuous stirring at 150 rpm. After stirring for 30 min, 2 mL of cyanamide solution (50 wt.% in water, Sigma-Aldrich, St. Louis, MO, USA) as nitrogen source was added into the mixture and stirred for another 30 min at the same rate. Water was then removed by rotary evaporation, and the product was completely dried in an oven at 95 °C overnight. The dried solid was ground and pyrolyzed following a temperature program reported previously [10,40]. Finally, the products were washed with deionized water and then lyophilized overnight. The obtained materials were denoted as NG/CB-x, where x is the mass ratio of GO to CB, x = 1, 2, 5, 10, and 20. 

### 2.2. Electrochemical Analysis

The electrochemical measurements were conducted on a CHI 760E electrochemical workstation assembled with an MSR electrode rotator (Pine Instrument Co., Grove City, PA, USA). A typical three-electrode electrochemical reactor was used. Ag/AgCl (saturated KCl) and platinum mesh were used as the reference and counter electrodes, respectively. A 5.0 mm diameter glassy carbon disk (Pine Instrument Co., Grove City, PA, USA) with Pt ring was used in the rotating ring-disk electrode (RRDE) experiments.

The catalyst ink was made by ultrasonically dispersing 5.0 mg of NG/CB-x or 20 wt.% Pt/C (HISPEC 3000, Johnson Matthey Corp., London, UK) in 5 mL of ethanol and 50 μL of 5 wt.% Nafion (DuPont Company). Approximately 19.6 μL of the ink was carefully dripped onto the glassy carbon, which was initially polished using a 0.05 mm alumina slurry. The loading of all catalysts on the GC was 100 μg·cm^−2^.

All the RRDE tests were carried out at 25 °C in N_2_- or O_2_-saturated 0.1 M phosphate buffered saline (PBS, pH 7.0) aqueous solution. Tests of linear sweep voltammetry (LSV) at a rotation rate of 1600 rpm were performed. The disk potential varied from −0.6 V to 0.6 V (vs. Ag/AgCl) at a potential sweep rate of 5 mV·s^−1^. The ring potential was set at 0.6 V (vs. Ag/AgCl). The yield percentage of H_2_O_2_ and the electron-transfer number (*n*) were calculated using the following equations:

(1) $$\%H_{2}O_{2}=200\times \frac{I_{r}/N}{I_{d}+I_{r}/N}$$

(2) $$n=4\times \frac{I_{d}}{I_{d}+I_{r}/N}$$

where *I_d_* is the disk current, *I_r_* is the ring current, and *N* is the current collection efficiency of the Pt ring. *N* was determined to be 0.21 ± 0.01 from the reduction of K_3_Fe[CN]_6_.

Half-cell electrochemical measurements of air-cathode were carried out in a single-chamber MFC reactor on a 2273 Princeton workstation (Princeton Applied Research, Ametek Inc., Berwyn, PA, USA). A three-electrode electrochemical system was established, which contained a working electrode (prepared air-cathode), a platinum mesh (2 × 2 cm^2^) counter electrode, and a KCl saturated Ag/AgCl reference electrode in O_2_-saturated 0.1 M PBS electrolyte. LSV from 0.4 to −0.2 V (vs. Ag/AgCl) at a scan rate of 1 mV·s^−1^ was conducted. The potential at which one can observe a sharp increase in reduction current was defined as onset potential. The Tafel plot was recorded by sweeping the overpotential from 0 mV to 100 mV at 1 mV·s^−1^. The exchange current density (*j*_0_) was calculated form Tafel plots in the overpotential interval of 80 and 100 mV [41]. Electrochemical impedance spectroscopy (EIS) was conducted at 0.2 V in a frequency of 100 kHz to 10 mHz with an amplitude of 10 mV. The calibration of the Ag/AgCl reference electrode was carried out following a previous study [42], and all potentials were then converted to the reversible hydrogen electrode (RHE).

### 2.3. MFC Set-Up and Inoculation

The dual-chamber air-cathode MFCs (Phychemi Co., Ltd., HK, China, Figure S1) with a cylindrical chamber (lengths of 2 cm and diameters of 3 cm for each chamber) was made of plexiglass. Heat-treated carbon felt (Alfa Aesar, 2 cm × 2 cm × 0.2 cm) was used as the anode. An air-cathode made from waterproof carbon cloth (W1S1005, CeTech, Taiwan) containing poly(dimethylsiloxane) as a diffusion layer was fabricated as described in a previous study [43]. A pretreated proton-exchange membrane (Nafion^®^ 117, Dupont China Holding Co., LTd., Beijing, China) was used to separate the anode and cathode chambers. The loading of catalysts including NG/CB-x and Pt/C on air-cathode was 2.5 mg·cm^−2^, which were sprayed on the water-facing side of carbon cloth. The anode and cathode were connected with a 1000 Ω of an external resistor using titanium wire, and all exposed metal surfaces were sealed with nonconductive epoxy.

The reactors were inoculated with an aged solution obtained from an MFC that had been operating for over a year in the laboratory. The aged inoculation solution contains abundant active electricigens. The fresh anolyte was mainly constituted of sodium acetate (1 g·L^−1^) and 0.1 M PBS (pH 7.0), as well as KCl (0.13 g·L^−1^), NH_4_Cl (0.31 g·L^−1^), minerals (10 mL·L^−1^), and vitamins (10 mL·L^−1^). The ingredients of mineral and vitamin solutions followed previous reports [43,44]. The volume ratio of the aged inoculation solution to fresh anolyte of 1:10 was used during inoculation stage. After the completion of inoculation, only fresh anolyte was used. The catholyte was 0.1 M PBS only without any organic compound and other ingredients. The cell voltage across the external resistor was measured using a multimeter equipped with a data acquisition system (PCI-1747U, Advantech, Taiwan). All MFCs were operated at a constant temperature of 30 ± 1 °C. The feed solution was replaced when the cell voltage dropped to 20 mV. All of the experiments were conducted in duplicates to achieve the experimental data statistical soundness.

The power densities of the cells were acquired from the polarization curves at various external resistors from open circuit voltage (OCV) to 10 Ω as the performance of the MFC approached steady-state conditions. Data were recorded as <1 mV variation in voltage. During the tests, the electrode potentials of anode and cathode were synchronously recorded. Ag/AgCl reference electrodes were used and placed vertically parallel to anode and cathode, respectively. All of the experiments were conducted twice at 30 ± 1 °C. Power densities of the cells were calculated based on the cathode projected area as follows:

(3) $$p=\frac{E_{cell}^{2}}{A_{cat}R_{ext}}$$

where *E_cell_* is the cell voltage, *R_ext_* is the external resistance, and *A_Cat_* is the cathode projected area. Coulombic efficiency (CE) was calculated using Equation (4):

(4) $$\epsilon_{cb}=\frac{8\int_{0}^{t}Idt}{FV_{An}\Delta_{COD}}$$

where *V_An_* is the volume of liquid in the anode chamber, and Δ*_COD_* is the change in chemical oxygen demand (COD) over time *t*.

The CE is defined as the ratio of total coulombs actually captured by the MFC from the substrate to the maximum possible coulombs if all substrate produced current. The COD represented the relative content of organic matters, which was analyzed on a COD meter (DR3900, Hach, Loveland, CO, USA).

### 2.4. Characterization of NG/CB-x

The morphology of NG/CB-x was determined by scanning electronic microscopy (SEM; JSM-7800F, JEOL, Tokyo, Japan) and transmission electronic microscopy (TEM; Zeiss LIBRA 200 FE, Oberkochen, Germany). The crystal of the catalyst was characterized by X-ray diffraction (XRD) using Cu Kα radiation (40 kV and 40 mA) implemented with a D8 advance diffractometer (1.54056 Å, Bruker, Bruker Beijing Office, China). The 2*θ* ranged from 10° to 90° at a scan rate of 0.02° and a fixed acquisition time of 0.1 s. The defection degree of carbon was acquired using an inVia Raman microspectrometer (Renishaw, Wharton-Ander Edge, UK) at 532 nm. X-ray photoelectron spectra (XPS) were carried out on ESCALAB 250Xi (Thermo Fisher Scientific, Waltham, MA, USA) X-ray photoelectron spectroscope using monochromatic Al Kα radiation at 1486.6 eV. The data for each atom and band deconvolution were fitted with the ‘XPS peak’ software, and the C1s peak at 284.8 eV was used as an internal standard. The Brunauer-Emmett-Teller (BET) surface area was obtained from 77 K N_2_ sorption isotherms using a Belsorp-max instrument (MicrotracBEL, Osaka, Japan). Pore size distribution was calculated according to Barett–Joyner–Halenda (BJH) model, and the t-plot method was used to extract the microporous surface area and volume. 

## 3. Results and Discussion

### 3.1. Electrocatalytic Activity for ORR in Neutral-pH Medium

**Figure 2:**
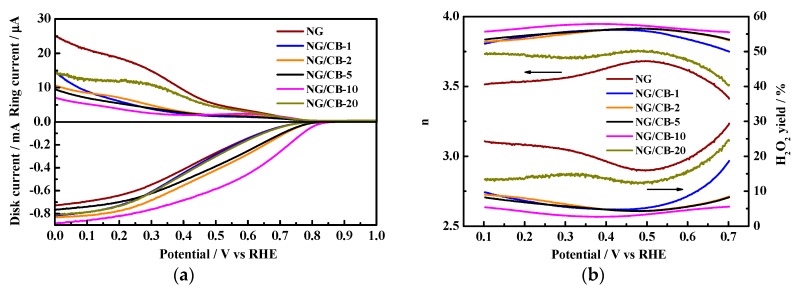
The current-potential profiles of RRDE tests in O_2_-saturated 0.1 M PBS at 1600 rpm (**a**) and corresponding H_2_O_2_ yield and electron transfer number (**b**) of NG/CB-x electrocatalysts. The electrocatalyst loading on RRDE was 100 μg cm^−2^.

CB addition into NG is expected to influence the electrochemical performance of NG/CB-x composites, which was investigated by means of RRDE tests. Figure 2a demonstrates the overlaid RRDE results of all NG/CB-x electrocatalysts. As clearly shown in this figure, the CB addition can evidently increase the ORR current and decrease the ring current in comparison with pristine NG. The result suggested that CB addition can expectedly improve the electrical conductivity of NG/CB-x composites. The decreased ring current of NG/CB-x composites was indicative of lesser H_2_O_2_ was formed during the process of oxygen reduction on disk electrode compared with NG. Therefore, the efficiency of ORR on NG/CB-x composites was enhanced in comparison with NG. Higher electron-transfer number of NG/CB-x composites than that of NG was also expected. The highest ORR current and lowest ring current relative to NG/CB-10 was observed, which illustrated that this electrocatalyst can catalyze ORR with the highest efficiency. The superior current performance of NG/CB-10 was possibly due to the abundant active sites for O_2_ adsorption and reduction.

Onset potential is an important factor representing the ORR ability of electrocatalyst. On the basis of LSV, the onset potentials for all electrocatalysts are summarized in Table 1. After the addition of CB, the onset potential showed a prominent positive shift from 0.798 V (vs. RHE) of NG to 0.864 V (vs. RHE) of NG/CB-10. A less positive shift in onset potential was observed for the electrocatalysts with excessive (0.805 V for NG/CB-1) or insufficient (0.798 V for NG/CB-20) CB addition to NG. The small quantity of CB in NG/CB-20 composite cannot adjust the electrochemical properties of pristine NG, whereas excessive CB might cover up the active sites of NG so that the ORR activity of NG/CB-1 was suppressed. 

Figure 2b shows the results of potential-dependent H_2_O_2_ yield and *n* number of NG/CB-x electrocatalysts, and the average values of *n* number and H_2_O_2_ yield are listed in Table 1. Undoubtedly, pristine NG exhibited 2e^−^ and 4e^−^ mixed ORR pathways in terms of *n* of 3.58 with H_2_O_2_ yield as high as 20.73%. The average *n* number of NG/CB-x electrocatalysts were 3.86, 3.87, 3.88, 3.92, and 3.71 for NG/CB-1, NG/CB-2, NG/CB-5, NG/CB-10, and NG/CB-20, respectively. This result was in accordance with that of RRDE. The lowest H_2_O_2_ yield (3.85%) of NG/CB-10 indicated that ORR on this composite proceeded through 4e^−^ reaction pathway, which can provide high electromotive force in MFCs when the material was applied as cathode electrocatalyst. Furthermore, the NG/CB-10 displayed comparable ORR activity to the state-of-the-art Pt/C in terms of *n* number and H_2_O_2_ yield despite the minimal current, as shown in Figure S2. However, the onset potential of NG/CB-10 was more negative than that of Pt/C. The results suggested that CB addition played an important role in the enhancement in ORR activity of NG.

Poor stability is a major problem that limits the application of NG. Restacking of graphene sheets reduces the surface area and the accessibility of active sites of NG. Therefore, the ORR activities of NG and NG/CB-10 before and after one-month storage in ambient condition were examined and compared in Figure S3. The current of stored NG was obviously reduced in comparison with fresh NG. By contrast, the current of NG/CB-10 showed little discrepancy before and after one-month storage. For instance, the disk current of NG at the potential of 0.2 V (vs. RHE) dramatically reduced from 0.64 mA to 0.39 mA. However, the current of NG/CB-10 at this potential only decreased from 0.82 mA to 0.78 mA. The *n* number of NG also decreased from 3.58 to 3.51 before and after storage. The *n* number of NG/CB-10 negligibly decreased from 3.92 to 3.91 in the same storage period. The result undoubtedly indicated that CB addition indeed enhanced the structure stability of NG and therefore prolonged the lifetime of NG/CB-10, making the catalyst suitable for practical application.

**Figure 3:**
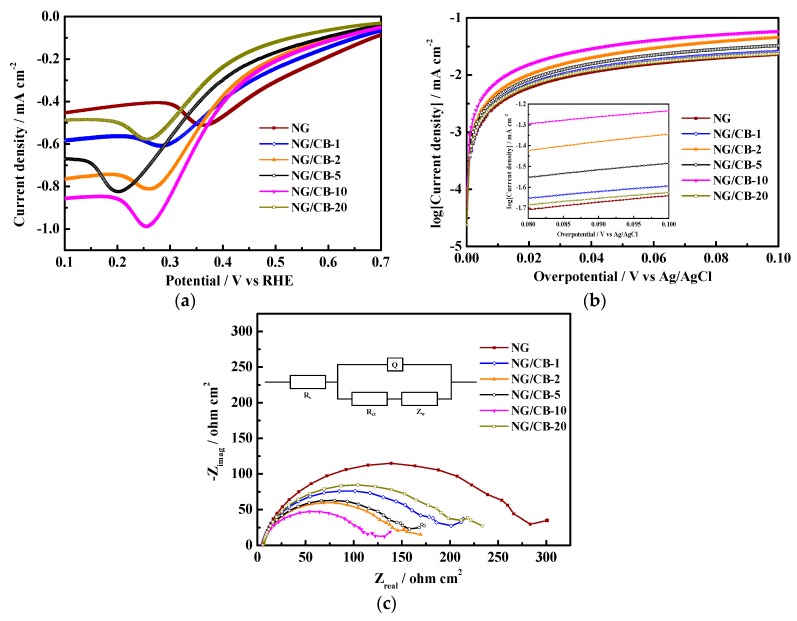
Half-cell tests of NG/CB-x electrocatalysts in O_2_-saturated 0.1 M PBS (electrocatalyst loadings of NG/CB-x were 15 mg cm^−2^): LSV curves (**a**) Tafel plots ((**b**) inset: linear fitting in the overpotential interval of 80 and 100 mV), and EIS tests ((**c**) inset: equivalent circuit).

Half-cell tests were carried out to further investigate the electrochemical performance of NG/CB-x as electrocatalysts on air-cathode. The overlaid LSV curves in Figure 3a display obvious oxygen reduction peaks for all NG/CB-x composites. Moreover, the peak current densities of all NG/CB-x composites were higher than that of NG, which was in agreement with the result of RRDE tests. The NG/CB-10 showed almost twice higher peak current density than that of NG. The results of LSV revealed that the ORR activity of NG was ultimately enhanced by CB addition as support and current collector. The Tafel plots were applied to evaluate the ORR kinetics of NG/CB-x composites, and the results are shown in Figure 3b. The value of *j*_0_ was obtained by conducting linear fitting in the overpotential interval of 80 and 100 mV (inset of Figure 3b). As listed in Table 1, the *j*_0_ of NG/CB-10 was the highest among all composites, which was 168% higher than that of NG. A high value of *j*_0_ was responsible for rapid ORR kinetics and low activation barrier. The results of Tafel behavior were in agreement with the findings of RRDE and LSV. The order of *j*_0_ value was in agreement with the order of *n* number. The improved ORR kinetics of NG/CB-x composites is suggested to facilitate the complete conversion of O_2_ into H_2_O by enhancing electron transfer. 

As a powerful tool, EIS was also used to evaluate the electrochemical properties of air-cathodes loaded with different electrocatalysts. The Nyqusit plots of all electrocatalysts are overlaid in Figure 3c, and an equivalent circuit (inset of Figure 3c) used for data fitting indicated that the cathode reaction was affected by reaction kinetics and mass transport [45,46]. As shown in Table 1, the values of electrolyte diffusion resistance (R_s_) of all EIS tests were almost identical due to the same configuration of single-chamber MFC reactor. However, the values of charge transfer resistance (R_ct_) for all NG/CB-x composites showed obvious differences. The R_ct_ significantly decreased by 52.1% from 226.7 Ω·cm^2^ (NG) to 108.7 Ω·cm^2^ (NG/CB-10). These findings indicated that NG/CB-10 can accelerate electron transfer and diminish the accumulation of intermediates of ORR, which resultantly led to the 4e^−^ ORR pathway on NG/CB-10. 

### 3.2. Characterization of NG/CB-x

**Figure 4:**
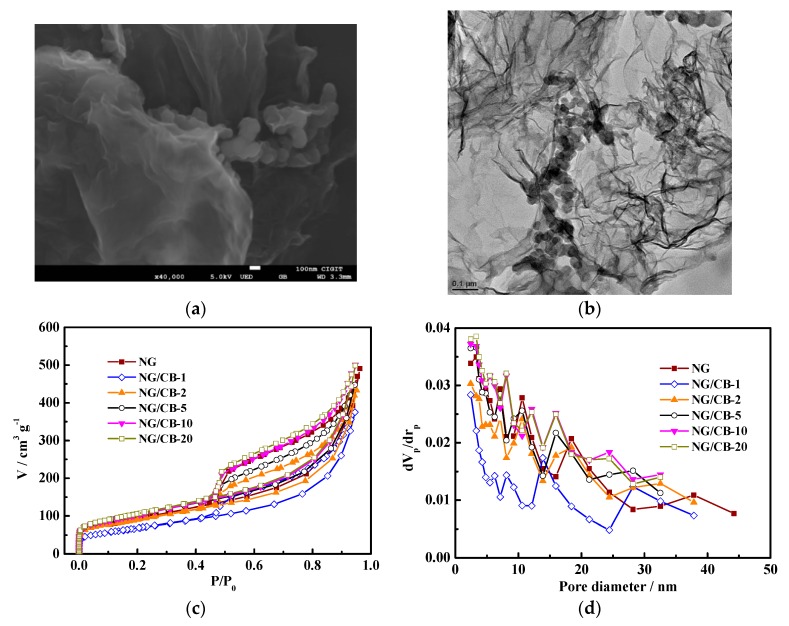
SEM (**a**) and TEM (**b**) images of NG/CB-10, and N_2_ adsorption and desorption isotherms (**c**) and pore size distribution curves (**d**) of NG/CB-x electrocatalysts.

Figure 4a and Figure S4 present the SEM images of NG/CB-x composites as well as NG and CB. The pristine NG (Figure S4a) displayed typical thin graphene nanolayers with porous and wrinkled structure, which was attributed to the incorporation of nitrogen into the graphene lattice [47]. The morphologies of NG/CB-x composites were clearly dependent on the mass percentage of CB. At high CB content, the morphology of NG/CB-1 was identical to that of CB, which was possibly due to the small mass percentage of NG in the composite. However, at low CB content, the morphology of NG/CB-20 was almost the same with that of NG, in which a few CB particles were encapsulated by thin graphene nanolayers. The TEM images in Figure 4b and Figure S5 also displayed the same tendency of NG/CB-x composites. The NG/CB-10 possessed an ideal NG encapsulated CB structure, in which the CB particles gathered inside and wrinkled NG nanolayers formed outside. This structure can expose abundant active sites and facilitate the ORR on NG/CB-10.

The effects of CB addition on surface area and porosity of NG were further investigated through N_2_ adsorption–desorption experiments. As shown in Figure 4c, an obvious hysteresis loop was observed on N_2_ adsorption–desorption isotherm curve for each material, which indicated that all NG/CB-x composites possessed mesoporous structure. Furthermore, the rapid increase of N_2_ adsorption–desorption isotherm curve of at low relative pressure indicated the presence of micropores [20,25]. The BJH pore size distribution curves in Figure 4d confirmed the existence of abundant mesopores due to the observation of pore size <10 nm for all samples. Table 2 lists the pore textures of all NG/CB-x composites, and the obvious effect of CB addition on the surface area was observed. As the CB content increased, the BET surface area of NG/CB-x composites decreased from 382.91 m^2^·g^−1^ (NG/CB-20) to 252.58 m^2^·g^−1^ (NG/CB-1). The BET surface area of NG/CB-x composites was equal to that of 3D graphene nanosheets obtained from sacrificial support method [48]. With respect to NG/CB-10, the BET surface area increased by 12% compared with pristine NG. Nevertheless, the BET surface area of NG/CB-1 decreased by 25% in comparison with the NG. The result was in agreement with that of electronic microscopy. Appropriate content of CB is suggested to facilitate the prevention of the NG nanosheets from restacking and increasing the surface area. Furthermore, the NG/CB-10 possessed the highest portion of mesopores (>86%) among all composites. Micropores can provide sufficient activity sites for ORR of electrocatalysts [49], and mesopores enable efficient mass transport [50]. Therefore, the mass transport of NG/CB-10 can be enhanced, which was responsible for the improved ORR activity. 

**Figure 5:**
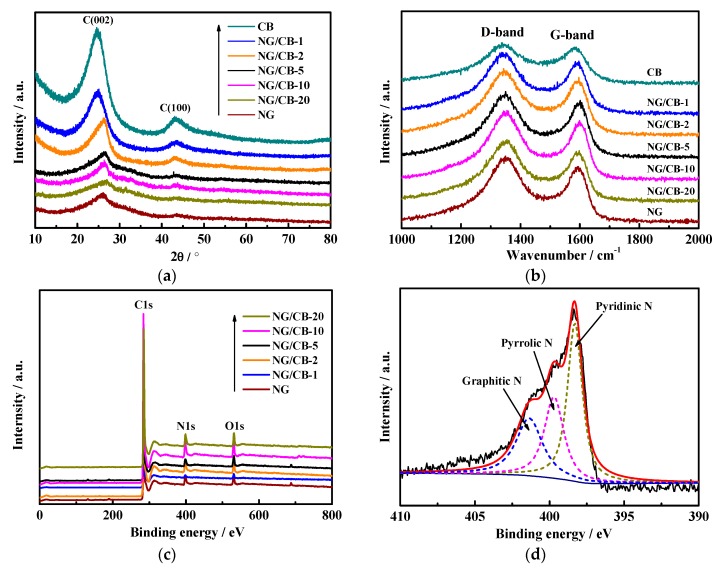
XRD (**a**), Raman (**b**) spectra and XPS survey (**c**) of NG/CB-x electrocatalysts, and high resolution of N1s of NG/CB-10 (**d**).

The crystalline structure of NG/CB-x electrocatalysts was investigated using XRD and Raman analyses, and the corresponding results are presented in Figure 5a,b. As depicted in Figure 5a, two apparent peaks around 26° and 43° were observed for all carbon materials, which corresponded to the (002) and (100) facets of disordered carbon, respectively [25]. With the increase of CB content, the peak of C (002) shifted to a low angle, peak width decreased, and peak intensity increased, corresponding to the increase in graphitization [51]. The Raman spectra (Figure 5b) demonstrated typical D and G bands at around 1350 and 1585 cm^−1^ for all composites. The G band was associated with the E2g mode of graphitic carbon, and the D band corresponded with the defect-induced mode, which resulted from the possible amorphous carbon with doped heteroatoms [11,52]. The ratio of intensities of the D and G bands (I_D_/I_G_) is often applied to estimate the average graphitization degree of different electrocatalysts, which were calculated and listed in Table 2. With the increase of CB content, the I_D_/I_G_ value decreased gradually, demonstrating the increase in average graphitization degree. This result from Raman spectra was identical to that of XRD. High graphitization degree often results in superior electrical conductivity and thus enhances the electrochemical performance of materials. Consequently, all NG/CB-x electrocatalysts possessed higher current densities and better ORR performance than pristine NG, as demonstrated in electrochemical measurements.

XPS was applied to determine the elemental compositions of NG/CB-x electrocatalysts. The survey spectra in Figure 5c indicate the presence of C 1s, N 1s, and O 1s for all NG/CB-x composites. The corresponding atomic percentage of each element is listed in Table 2. The NG/CB-10 contained a higher atomic percentage of O 1s than other NG/CB-x electrocatalysts. Some researchers discovered that oxygen-containing functional groups can serve as the active sites for O_2_ adsorption and electron transfer, which benefited the proceeding of ORR in a neutral-pH medium [10,53], indicating that NG/CB-10 possibly possessed excellent electrocatalytic activity. Furthermore, the NG/CB-10 contained the highest atomic percentage of N 1s among all materials. The active centers of the ORR are N-containing functional groups for nitrogen-doped carbon materials. As shown in Figure 5d and Figure S6, the high-resolution N 1s spectra for NG/CB-x composites can be deconvoluted into three peaks, including pyridinic nitrogen (398.2 eV), pyrrolic nitrogen (399.4 eV), and graphitic nitrogen (401.0 eV). Table 2 lists the atomic weight of each type of N species. The sum of pyridinic N (3.37 at.%) and pyrrolic N (2.26 at.%) for NG/CB-10 was higher than that of other materials, which was possibly ascribed to the GO encapsulated CB structure, and benefited the doping of nitrogen atoms into the nearby carbon lattice to form high content of pyridinic N and pyrrolic N [54]. Pyridinic N and pyrrolic N can decrease the adsorption energy of O_2_, resulting in the ORR occurring readily on N-doped carbon materials [55,56]. Therefore, the high content of pyridinic and pyrrolic N of NG/CB-10 contributed to the improved electrocatalytic activity. In recent research, Guo et al. pointed out that the carbon atoms next to pyridinic N with Lewis basicity might be the active sites for ORR [57]. In consequence, the highest content of pyridinic N in NG/CB-10 could generate the most number of carbon atoms with Lewis basicity, resulting in the best ORR performance among the NG/CB-x electrocatalysts.

### 3.3. MFC Performance Using Different Electrocatalysts

**Figure 6:**
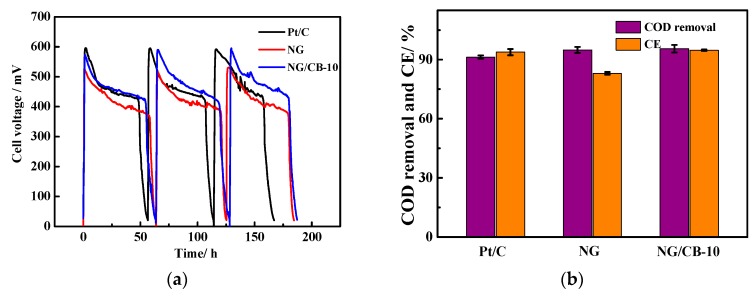

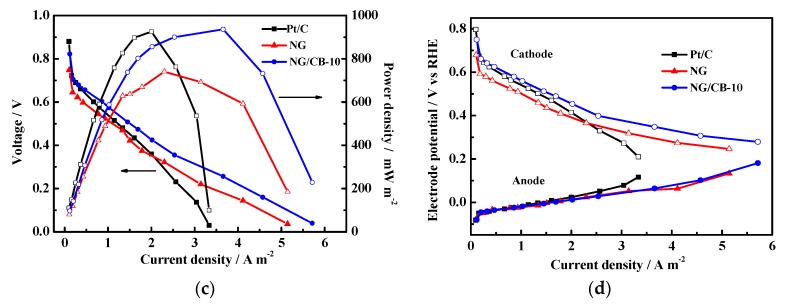
The voltage-time profile (**a**) external resistance of 1000 Ω and temperature of 30 ± 1 °C, COD removal and coulombic efficiency (**b**), polarization and power density curves (**c**) and electrode potentials (**d**) of MFCs equipped with different cathode electrocatalysts.

Pristine NG and NG/CB-10 along with commercial Pt/C were used as cathode electrocatalysts at the same loading of 2.5 mg·cm^−2^ in dual-chamber air-cathode MFCs. As shown in Figure 6a, the maximum voltage of MFCs equipped with different electrocatalysts followed the order: Pt/C (593 mV) > NG/CB-10 (585 mV) > NG (523 mV). The order of maximum cell voltage was in agreement with that of ORR onset potential of these electrocatalysts. The COD removals of these MFCs were almost identical to each other (Figure 6b), indicating similar degradation efficiency of organic compounds. Nevertheless, the values of CE showed significant difference among these MFCs. The NG-MFC showed the lowest CE, whereas the Pt/C-MFC showed the highest value. The CE of NG/CB-10-MFC was almost identical to that of Pt/C-MFC. The difference in electrocatalytic activity of cathode was responsible for the difference of CE due to the same cell configuration, anode material, and inoculation procedures. The result suggested that the electrocatalytic activity of NG/CB-10 cathode was enhanced in comparison with NG cathode, which was in agreement with the result of electrochemical characterization. 

Power generation is one of the most significant evaluation parameters of MFC performance. The polarization and power density curves of the MFCs are shown in Figure 6c. As shown in the polarization curves, the MFC equipped with NG/CB-10 generated an OCV of 0.822 V, which was slightly lower than that of Pt/C (0.880 V) but higher than that of NG (0.748 V). The discrepancy in the value of OCV was ascribed to the different electrochemical performance of these electrocatalysts. CB addition to NG reduced the overpotential of air-cathode and resulted in high OCV. The maximum power density of MFC using NG/CB-10 displayed the highest value of 936 mW·m^−2^, which was 26% higher than that of pristine NG (740 mW·m^−2^) and comparable to that of Pt/C (925 mW·m^−2^). Furthermore, the cell internal resistance was obtained from power density curves showed the following order: NG/CB-10 < Pt/C < NG. CB addition is suggested to increase the conductivity of NG/CB-10 and improve electron transport, resulting in lower internal resistance. The electrode potentials shown in Figure 6d illustrated that the cathode performance played a critical role in the power generation of MFCs due to the identical anode potential curves among all electrocatalysts. The cathode potential at open circuit positively shifted from 0.68 V (vs. RHE) of NG to 0.75 V (vs. RHE) of NG/CB-10, thereby increasing the OCV of MFCs.

In early studies, NG has been verified to be an efficient ORR electrocatalyst and supercapacitor. However, cost and quality are among the most challenging obstacles that need to be overcome before the successful large-scale application of NG-based electrodes in MFCs [58]. The drawback of easy restacking of these electrodes hinders its practical application due to its considerably higher cost than activated carbon. The novel method proposed in the present work enables the successful synthesis of an NG/CB composite material. The modified composite possesses an enlarged surface area, enhanced ORR activity, and improved stability owing to the addition of conductive CB. Moreover, the price per gram of NG/CB-10 is cut down by 25% compared with NG. Without doubt, high performance and low price will facilitate NG/CB composite material to be practically utilized in MFCs as cathode electrocatalyst. Other properties of NG/CB composite material, especially anti-biofilm growth, will be further investigated.

## 4. Conclusions

To enhance the ORR activity and stability and reduce the cost of NG, the present work proposed a template-like method to fabricate NG/CB composite. The NG/CB composite possessed enlarged surface area, high graphitization degree, and high nitrogen content due to the addition of CB. Given these results, NG/CB-10 displayed the best electrochemical performance in neutral-pH medium, including onset potential, electron-transfer number, exchange current density, as well as charge transfer resistance. At the same catalyst loading, the MFC equipped with NG/CB composite displayed improved performance compared with pristine NG. The maximum power density of air-cathode MFC using NG/CB-10 as cathode electrocatalyst reached 936 mW·m^−2^, which was much higher than that of NG and comparable to Pt/C. These findings indicated that the template-like method was efficient to fabricate ORR electrocatalyst with low cost and high activity. The new composite will be promising alternative cathode electrocatalyst for the practical application of MFCs in the future.

## Figures and Tables

**Table 1:** Electrochemical properties of NG/CB-x electrocatalysts.

Samples	Onset Potential/V vs. RHE	*n*	H_2_O_2_ Yield/%	10^−2^ j_0_/mA·cm^−2^	R_s_/Ω·cm^2^	R_ct_/Ω·cm^2^
NG	0.798	3.58 ± 0.06	20.73 ± 3.15	1.06	5.85	226.7
NG/CB-1	0.805	3.86 ± 0.04	7.35 ± 3.00	1.30	5.56	177.4
NG/CB-2	0.832	30.87 ± 0.03	6.23 ± 1.50	1.83	5.75	138.2
NG/CB-5	0.836	3.88 ± 0.02	5.81 ± 1.16	1.52	5.83	144.8
NG/CB-10	0.864	3.92 ± 0.02	3.85 ± 0.93	2.85	5.38	108.7
NG/CB-20	0.798	3.71 ± 0.05	14.70 ± 2.52	1.20	5.79	200.8

**Table 2:** Characteristics and textural parameters of NG/CB-x electrocatalysts.

Parameters	NG	NG/CB-1	NG/CB-2	NG/CB-5	NG/CB-10	NG/CB-20
SBET/m^2^·g^−1^	336.62	252.58	331.14	371.52	376.94	382.91
Smicropore/m^2^·g^−1^	43.73	14.47	71.8	83.14	52.54	59.49
Smesopore/m^2^·g^−1^	292.89	238.11	259.34	288.38	324.40	323.42
Total Vpore/cm^3^·g^−1^	0.76	0.58	0.67	0.69	0.77	0.77
Vmicropore/cm^3^·g^−1^	0.07	0.03	0.05	0.06	0.07	0.08
Vmesopore/cm^3^·g^−1^	0.69	0.55	0.62	0.63	0.70	0.69
Mean pore diameter/nm	9.02	6.73	8.09	7.35	8.05	8.34
ID/IG	1.17	1.04	1.05	1.08	1.09	1.14
C1s/at.%	88.80	93.80	90.27	89.64	87.80	88.55
N1s/at.%	6.57	3.14	5.95	6.34	7.79	7.19
Pyridinic N	2.63 (40.02%) ^a^	1.11 (35.39%)	2.56 (43.05%)	2.13 (33.64%)	3.37 (43.26%)	3.15 (43.78%)
Pyrrolic N	1.95 (29.67%)	1.00 (31.73%)	1.70 (28.51%)	2.04 (32.17%)	2.26 (28.99%)	2.03 (28.18%)
Graphitic N	1.99 (30.31%)	1.03 (32.88%)	1.69 (28.44%)	2.17 (34.19%)	2.16 (27.75%)	2.01 (28.03%)
O1s/at.%	4.63	3.06	3.78	4.02	4.41	4.26

^a^ The relative percentage of each N functionality among the N content on the composite surface.

## Supplementary Materials

The following are available online at https://www.mdpi.com/2079-4991/9/6/836/s1, Figure S1. The schematic figure of dual-chamber microbial fuel cell, Figure S2: The current-potential profiles of RRDE tests in O_2_-saturated 0.1 M PBS at 1600 rpm (a) and corresponding H_2_O_2_ yield and electron transfer number (b) of Pt/C, Figure S3: The current-potential profiles of RRDE tests in O_2_-saturated 0.1 M PBS at 1600 rpm (a) and corresponding H_2_O_2_ yield and electron transfer number (b) of NG and NG/CB-10 electrocatalysts before and after one-month storage, Figure S4: SEM images of NG (a), NG/CB-1 (b), NG/CB-2 (c), NG/CB-5 (d), NG/CB-20 (e) and CB (f), Figure S5: TEM images NG (a), NG/CB-1 (b), NG/CB-2 (c), NG/CB-5 (d), NG/CB-20 (e) and CB (f), Figure S6: High resolution of N1s of NG (a), NG/CB-1 (b), NG/CB-2 (c), NG/CB-5 (d) and NG/CB-20 (e).
