# Supplementary material for: Novel Porous Nitrogen Doped Graphene/Carbon Black Composites as Efficient Oxygen Reduction Reaction Electrocatalyst for Power Generation in Microbial Fuel Cell
Source: Nanomaterials (Basel). 2019 Jun 1;9(6):836. doi: 10.3390/nano9060836 (PMC6631044; doi:10.3390/nano9060836)
Supplement: Supplementary file 1 [file nanomaterials-09-00836-s001.pdf]

Supplementary Materials

Novel porous nitrogen doped graphene/carbon black composites as efficient oxygen reduction reaction electrocatalyst for power generation in microbial fuel cell

Yuan Liu [1,2,*], Zhimei Liu [2], Hong Liu [1,2,*] and Meiling Liao [1,2]

1 Key Laboratory of Reservoir Aquatic Environment, Chinese Academy of Sciences, China; liaomeiling@cigit.ac.cn (M.L.)
2 Chongqing Institute of Green and Intelligent Technology, Chinese Academy of Sciences, Chongqing 400714, China; zmliu@cigit.ac.cn (Z.L.)
* Correspondence: liuyuan@cigit.ac.cn (Y.L.); liuhong@cigit.ac.cn (H.L.); Tel.: +86-23-65935802 (Y.L.); +86-23-65935802 (H.L.)

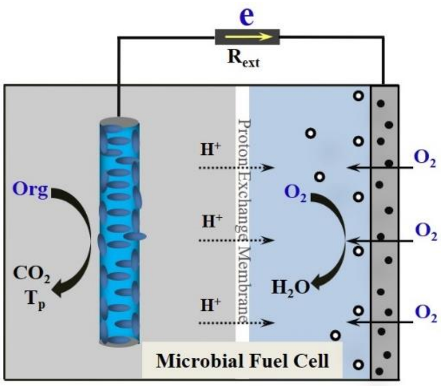

Figure S1. The schematic figure of dual-chamber microbial fuel cell.

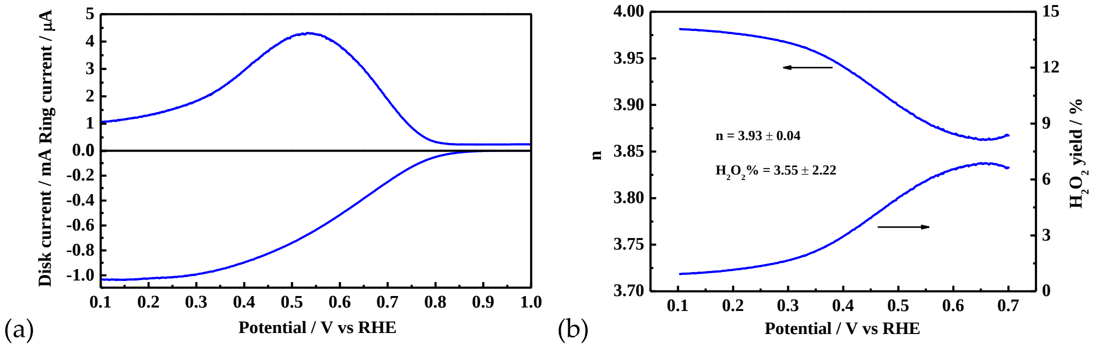

Figure S2. The current-potential profiles of RRDE tests in O_2-saturated 0.1 M PBS at 1600 rpm (a) and corresponding H_2O_2 yield and electron transfer number (b) of Pt/C.

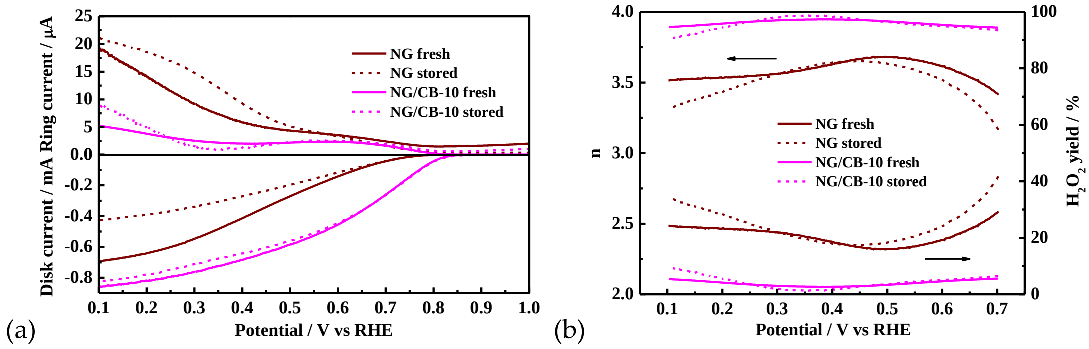

Figure S3. The current-potential profiles of RRDE tests in O_2-saturated 0.1 M PBS at 1600 rpm (a) and corresponding H_2O_2 yield and electron transfer number (b) of NG and NG/CB-10 electrocatalysts before and after one-month storage.

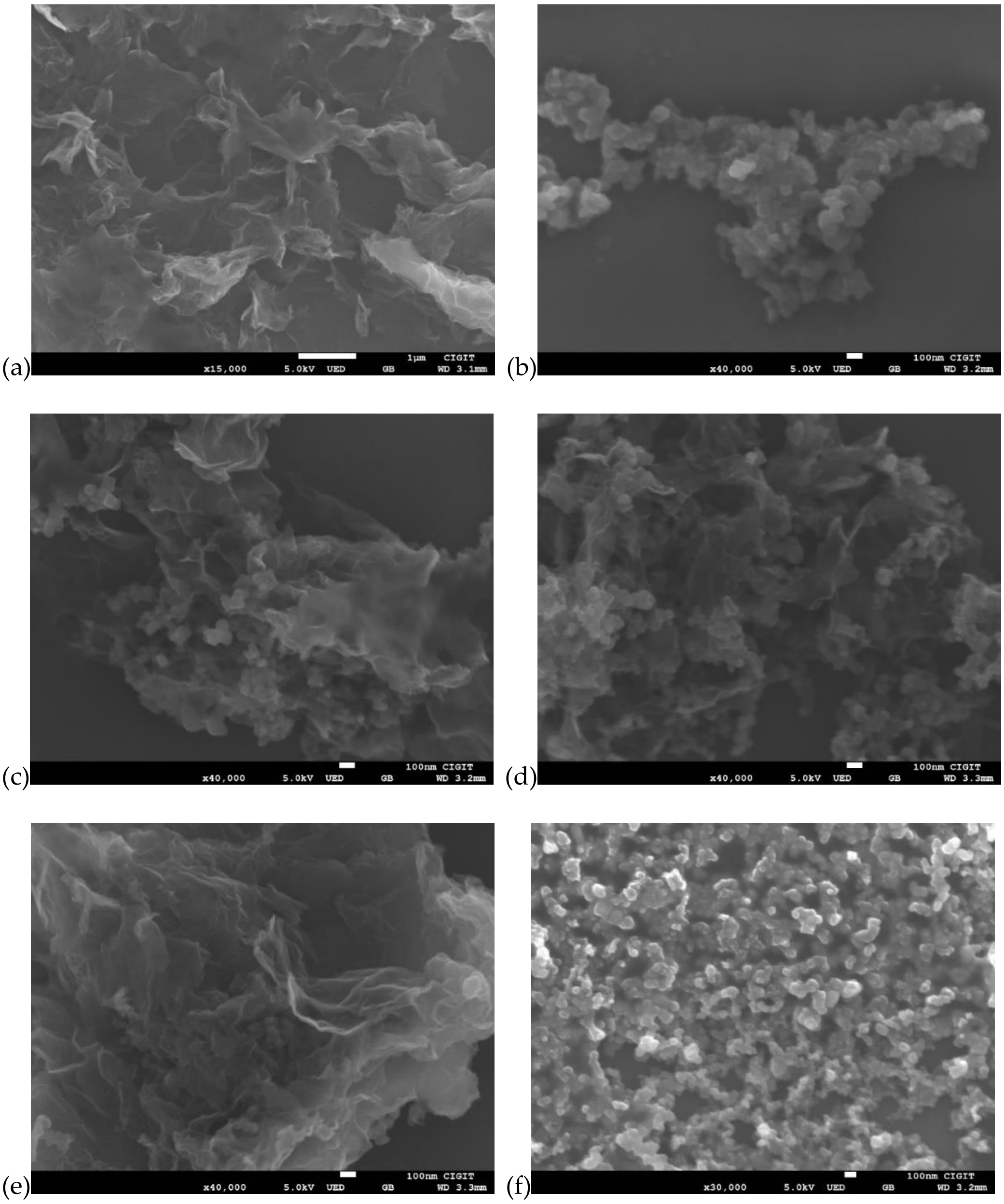

Figure S4. SEM images of NG (a), NG/CB-1 (b), NG/CB-2 (c), NG/CB-5 (d), NG/CB-20 (e) and CB (f).

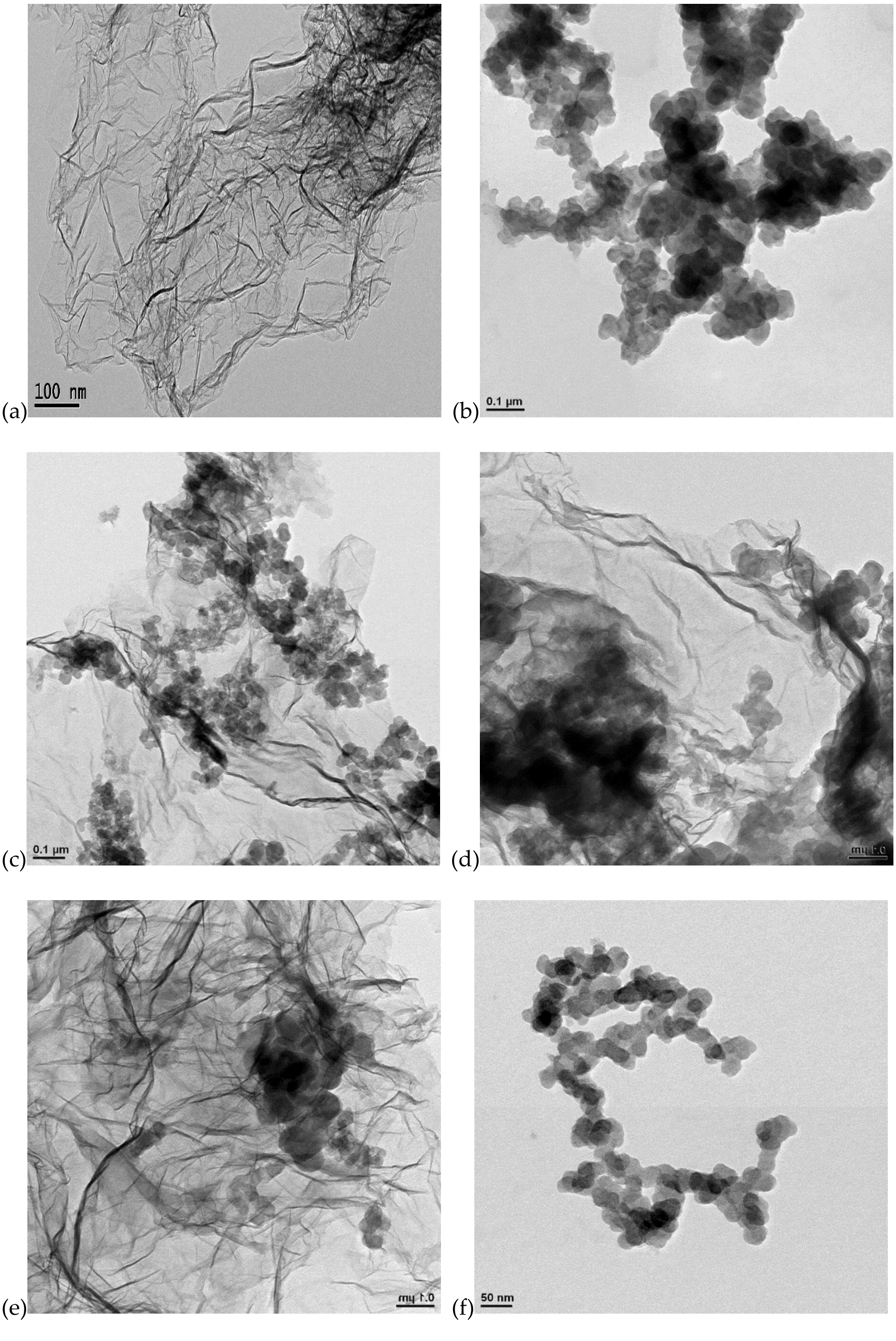

Figure S5. TEM images NG (a), NG/CB-1 (b), NG/CB-2 (c), NG/CB-5 (d), NG/CB-20 (e) and CB (f).

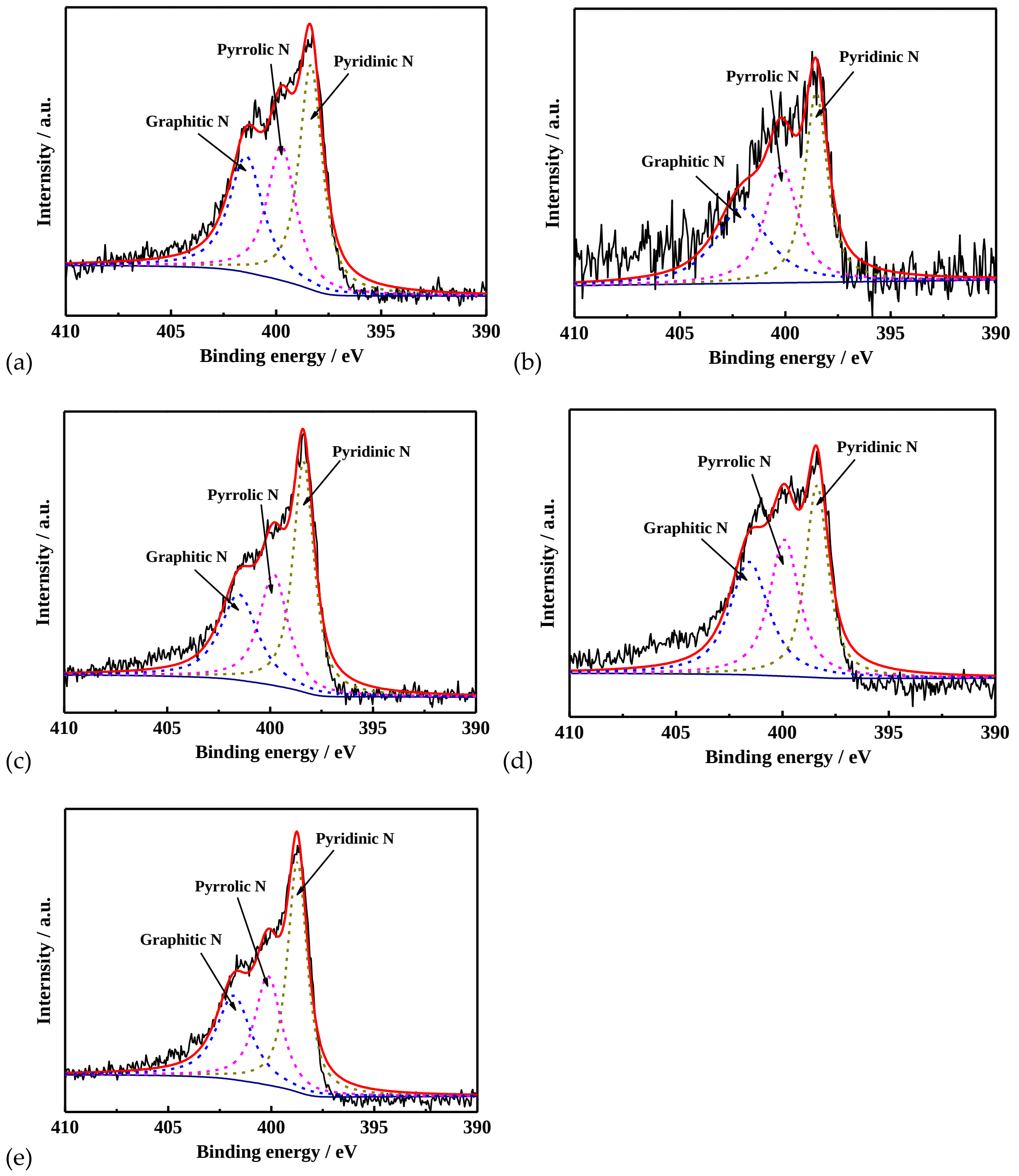

Figure S6. High resolution of N1s of NG (a), NG/CB-1 (b), NG/CB-2 (c), NG/CB-5 (d) and NG/CB-20 (e).
